# EGF Signal Propagation during C. elegans Vulval Development Mediated by ROM-1 Rhomboid

**DOI:** 10.1371/journal.pbio.0020334

**Published:** 2004-09-28

**Authors:** Amit Dutt, Stefano Canevascini, Erika Froehli-Hoier, Alex Hajnal

**Affiliations:** **1**Zoologisches Institut, Universität ZurichZurichSwitzerland

## Abstract

During Caenorhabditis elegans vulval development, the anchor cell (AC) in the somatic gonad secretes an epidermal growth factor (EGF) to activate the EGF receptor (EGFR) signaling pathway in the adjacent vulval precursor cells (VPCs). The inductive AC signal specifies the vulval fates of the three proximal VPCs P5.p, P6.p, and P7.p. The C. elegans Rhomboid homolog ROM-1 increases the range of EGF, allowing the inductive signal to reach the distal VPCs P3.p, P4.p and P8.p, which are further away from the AC. Surprisingly, ROM-1 functions in the signal-receiving VPCs rather than the signal-sending AC. This observation led to the discovery of an AC–independent activity of EGF in the VPCs that promotes vulval cell fate specification and depends on ROM-1. Of the two previously reported EGF splice variants, the longer one requires ROM-1 for its activity, while the shorter form acts independently of ROM-1. We present a model in which ROM-1 relays the inductive AC signal from the proximal to the distal VPCs by allowing the secretion of the LIN-3L splice variant. These results indicate that, in spite of their structural diversity, Rhomboid proteins play a conserved role in activating EGFR signaling in *C. elegans, Drosophila,* and possibly also in mammals.

## Abstract 2

ROM-1 increases the range of EGF, which specifies the vulval fate of precursor cells, by functioning in the signal- receiving precursor cells rather than the signal-sending anchor cells

## Introduction

Intercellular signaling pathways control many diverse processes, such as cell proliferation, differentiation, survival, migration, shape changes, and responses to the environment. In most instances, the release of the signaling molecules by the signal-sending cells constitutes a rate-limiting step that determines the spatial distribution and temporal duration of the response (Freeman and Gurdon 2002). On the other hand, the binding sites on the receptors that are presented by the signal-receiving cells are usually in excess of the available ligands (Freeman and Gurdon 2002). Specificity is achieved by the tissue-specific expression of the signal or by the regulated activation of an inactive precursor molecule. For example, growth factor peptides are often produced as inactive precursors that need to be processed before they can be released and activate their cognate receptors on the signal-receiving cells (Arribas et al. 1996).

The epidermal growth factor (EGF) receptor (EGFR) acts in a highly conserved signal transduction pathway that controls various cell fate decisions in metazoans (Bogdan and Klambt 2001). EGFR ligands of the transforming growth factor-α (TGF-α) family are produced as membrane-tethered precursor proteins with a single extracellular EGF repeat that is cleaved off the membrane anchor (Pandiella and Massague 1991; Bosenberg et al. 1993). The best-studied example of EGF processing is probably the *Drosophila* growth factor Spitz, which activates the EGFR in multiple developmental processes (Rutledge et al. 1992; Golembo et al. 1996). Genetic analysis of *Drosophila* EGFR signaling has identified Rhomboid-1 as a protein necessary for Spitz activation in the signal-sending cell (Bier et al. 1990; Golembo et al. 1996; Guichard et al. 2000; Wasserman et al. 2000). *Drosophila* Rhomboid-1 is the founding member of a family of seven-pass transmembrane proteins that function as intramembrane serine proteases (Urban et al. 2001). Site-specific cleavage of Spitz by Rhomboid-1 in the Golgi apparatus allows the secretion of the extracellular portion of Spitz by the signal-sending cell (Lee et al. 2001; Urban and Freeman 2003). The *Drosophila* genome encodes a total of seven *rhomboid* genes with partially overlapping functions in different tissues that utilize the EGFR pathway (Wasserman et al. 2000; Urban et al. 2002). There are four predicted Rhomboid homologs in humans and five in C. elegans (Wasserman et al. 2000). Rhomboid-like proteins are even found in yeast and bacteria (Gallio et al. 2002; McQuibban et al. 2003). Rhomboids are part of the larger family of I-Clip proteases that includes the aspartyl protease Presenilin (Wolfe et al. 1999) and the Zn^2+^ metalloprotease S2P (Urban and Freeman 2002). On the other hand, the secretion of vertebrate TGF-α involves processing by a disintegrin metalloprotease (ADAM), TNF-α-converting enzyme (Peschon et al. 1998). Whether a Rhomboid protease is also involved in the processing of TGF-α is currently unknown.

The development of the C. elegans hermaphrodite vulva serves as a simple model by which to study signal transduction and cell fate determination during organogenesis (Kornfeld 1997; Sternberg and Han 1998). During C. elegans postembryonic development, the anchor cell (AC) in the somatic gonad induces three out of six equivalent vulval precursor cells (the VPCs, termed P3.p through P8.p) in the ventral hypodermis to adopt vulval cell fates (Sulston and White 1980; Kimble 1981). The AC produces the LIN-3 growth factor, which is similar to *Drosophila* Spitz and mammalian TGF-α (Hill and Sternberg 1992). The VPCs express the EGFR homolog LET-23 on the basolateral surface that faces the AC (Whitfield et al. 1999), and they are all competent to activate the RAS/mitogen-activated protein kinase (MAPK) signaling pathway in response to the inductive LIN-3 EGF signal. The VPC closest to the AC, P6.p, adopts the primary (1°) cell fate characterized by a symmetrical cell lineage leading to eight 1° vulval cells (Sternberg and Horvitz 1986). The neighbors of P6.p, P5.p and P7.p, adopt the secondary (2°) cell fate, which is characterized by an asymmetrical lineage leading to seven 2° vulval cells. The more distally located VPCs, P3.p, P4.p, and P8.p, adopt the uninduced tertiary (3°) cell fate. After dividing once, they fuse with the surrounding hypodermal syncytium (hyp7). LIN-3 EGF dosage experiments have suggested that the inductive signal acts in a graded manner (Katz et al. 1995). According to this model, P6.p receives the highest amount of the inductive signal and thus adopts the 1° fate, while an intermediate level of the LIN-3 signal specifies the 2° fate in P5.p and P7.p. The distal VPCs, P3.p, P4.p, and P8.p, receive too little signal to adopt vulval cell fates. However, in response to the inductive signal, P6.p produces a lateral signal that activates the LIN-12 NOTCH signaling pathway in the neighboring VPCs, P5.p and P7.p (Greenwald et al. 1983). The LIN-12 NOTCH signal is both necessary and sufficient to induce the 2° cell fate (Sternberg 1988; Simske and Kim 1995). Thus, the graded LIN-3 EGF signal may act redundantly with the lateral LIN-12 NOTCH signal to specify the 2° vulval cell fate in the neighbors of P6.p (Kenyon 1995).

Like its *Drosophila* and vertebrate homologs, LIN-3 EGF is synthesized as a transmembrane precursor protein (Hill and Sternberg 1992). Experiments with *dig-1* mutants in which the AC is dorsally displaced indicate that the AC is capable of inducing vulval cell fates from a distance, suggesting that proteolytic cleavage of membrane-bound LIN-3 occurs in the AC (Thomas et al. 1990). Here, we report the identification of the C. elegans Rhomboid homolog ROM-1 as a positive regulator of vulval induction. Surprisingly, we find that ROM-1 acts in the signal-receiving VPCs rather than in the signal-sending AC. Furthermore, we uncover an AC-independent function of LIN-3 EGF that depends on ROM-1 activity in the VPCs. Two LIN-3 splice variants, termed LIN-3S and LIN-3L, that differ by an insertion of 15 amino acids in the region in the juxtamembrane domain critical for processing, have been described (Hill and Sternberg 1992). Genetic epistasis experiments indicate that LIN-3L activity in the VPCs depends on ROM-1, while LIN-3S or a truncated form of LIN-3 lacking the transmembrane domain act independently of ROM-1. We propose a relay model in which ROM-1 is required for the activation of LIN-3L in the proximal VPCs to transmit the inductive AC signal to the distal VPCs.

## Results

### Five Rhomboid-Like Proteins in C. elegans

**Figure 1:**
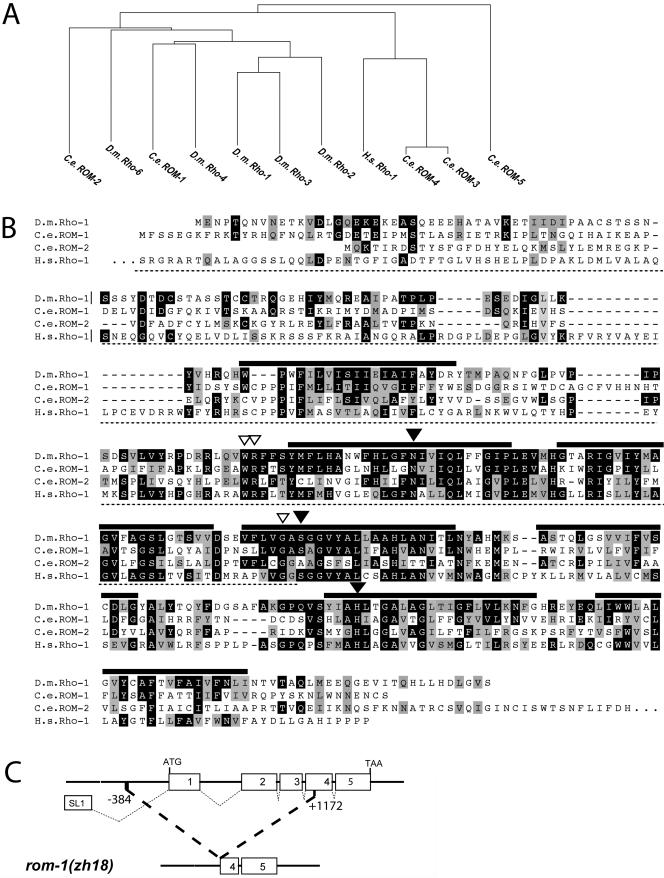
The C. elegans Rhomboid Genes (A) Dendogram showing the relation between the seven-pass transmembrane domains of Rhomboids from C. elegans (C.e.), Drosophila melanogaster (D.m.), and Homo sapiens (H.s.) calculated with the neighbor joining method using CLUSTAL X (Thompson et al. 1997). (B) Alignment of C. elegans (C.e.) ROM-1 and ROM-2 and Homo sapiens (H.s.) Rho-1 relative to Drosophila melanogaster (D.m.) Rho-1. Residues identical to those of *Drosophila* Rho-1 are highlighted in black, and similar residues are highlighted in grey. The thick black lines indicate the predicted seven-pass transmembrane domains. The three black triangles point at the residues forming a catalytic triad that forms a charge-relay system to activate the essential serine residue during peptide bond cleavage, and the three open triangles indicate other conserved residues necessary for the enzymatic activity as identified in D.m. Rho-1 (Urban et al. 2001). The region underlined with a dotted line indicates the extent of deletion in the *rom-1*(*zh18*) allele. (C) Intron-exon structure of the *rom-1* locus and extent of the deletion in the *rom-1(zh18)* strain. The numbers indicate the position of the deletion break-points relative to the A in the ATG start codon.

Since the LIN-3 EGF growth factor is produced as a transmembrane precursor protein (Hill and Sternberg 1992), we asked whether an intramembrane serine protease of the Rhomboid family is involved in the proteolytic processing of LIN-3 EGF. Rhomboid proteins in metazoans share a characteristic secondary structure consisting of seven transmembrane domains (Bier et al. 1990; Urban et al. 2001). We searched the complete C. elegans genome sequence for genes with similarity to *Drosophila rhomboid-1* and identified five *rhomboid-*like genes termed *rom-1* (F26F4.3), *rom-2* (C48B4.2), *rom-3* (Y116A8C.14), *rom-4* (Y116A8C.16), and *rom-5* (Y54E10A.14) (Figure 1A). All five C. elegans ROM proteins display the typical secondary structure of Rhomboids (Wasserman et al. 2000). The transmembrane domains show the highest degree of sequence conservation, while the hydrophilic N termini are more divergent (Figure 1B). ROM-1 is most similar to *Drosophila* Rhomboid-1 (35% identity), followed by ROM-2 (29% identity) and the more diverged ROM-3 (24% identity), ROM-4 (26% identity), and ROM-5 (29% identity). Mutagenesis experiments with *Drosophila* Rhomboid-1 have identified a catalytic triad formed by conserved asparagine, serine, and histidine residues that are necessary for the serine protease activity (Urban et al. 2001). This catalytic triad is conserved only in ROM-1 (black triangles in Figure 1B), suggesting that the other four Rhomboid-like proteins do not function as serine proteases*.*

In order to confirm the predicted intron-exon structure of *rom-1,* we isolated *rom-1* cDNA by RT-PCR. An SL1 trans-spliced leader sequence was identified at the 5′ end of the message that was spliced to the second of the six exons predicted by the C. elegans genome project (Figure 1C) (see http://www.wormbase.org). The remaining intron-exon boundaries were confirmed experimentally and corresponded exactly to the predicted boundaries. The conceptual translation of the 1,071-bp open reading frame (ORF) predicts a protein of 356 amino acids, with very short stretches of hydrophilic amino acids between the seven-pass transmembrane domains, except for a longer loop consisting of 43 amino acids between the first and second transmembrane domains (see Figure 1B).

### ROM-1 and ROM-2 Are Not Essential for Normal Vulval Development

As a first step to examine the biological function of the *rom* genes, we used RNA interference (RNAi) to transiently knock down their expression (Fire et al. 1998; Fraser et al. 2000; Kamath et al. 2001). Double-stranded RNA derived from a 352-bp *rom-1* or a 718-bp *rom-2* cDNA fragment of the divergent N-terminal portion was injected into the hermaphrodite gonads, and vulval development was examined in the F1 progeny under Nomarski optics. No obvious vulval phenotype was observed when *rom-1* or *rom-2* RNAi was performed in a wild-type background. Also, feeding wild-type or *let-60(n1046gf)* animals with bacteria producing *rom-3* dsRNA had no effect on vulval development (unpublished data). Due to the high degree of sequence similarity between *rom-3* and *rom-4* (69.8% identity), *rom-3* RNAi is likely to simultaneously reduce *rom-4* function.

Using a PCR-based assay to screen a library of mutagenized worms, we isolated a 1,556-bp deletion in the *rom-1* gene (see Figure 1C) (Jansen et al. 1997; Berset et al. 2001). The *zh18* deletion removes 206 amino acids from the N terminus, including the first three transmembrane domains and 384 bp of promoter sequences. Thus, the *zh18* deletion probably results in a complete loss of *rom-1* function and will be referred to as *rom-1(0)*. The *rom-1(0)* single mutants exhibited no obvious phenotype; they were healthy and fertile. In addition, we obtained the *rom-2(ok966)* allele from the C. elegans Gene Knockout Consortium. The *rom-2(ok966)* animals carry a 530-bp deletion that removes the fifth exon, which contains the predicted catalytic center with the essential histidine residue (see Figure 1B) (Urban et al. 2001). Since this allele is predicted to inactivate any potential protease activity of ROM-2, we refer to it as *rom-2(rf)*. Consistent with the RNAi experiments, both *rom-1(0)* and *rom-2(rf)* single mutants exhibited normal vulval development (Table 1, rows 2 and 3). Also, in *rom-1(0) rom-2(rf)* double mutants, no defects in vulval development were observed, ruling out a possible redundant function of the two genes (Table 1, row 4). Thus, neither ROM-1 nor ROM-2 are required for vulval induction under normal conditions.

**Table 1:**
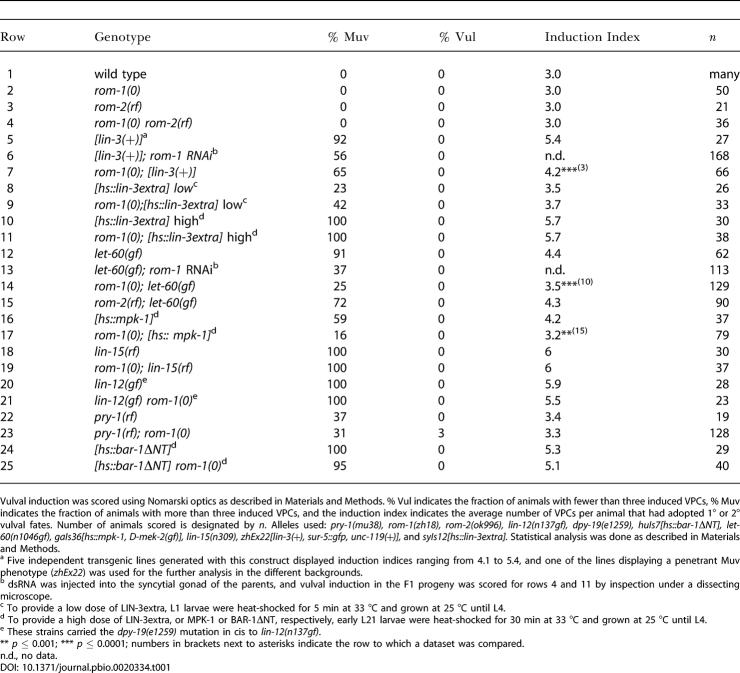
Suppression of Multivulva Mutants by *rom-1(0)*

Vulval induction was scored using Nomarski optics as described in Materials and Methods. % Vul indicates the fraction of animals with fewer than three induced VPCs, % Muv indicates the fraction of animals with more than three induced VPCs, and the induction index indicates the average number of VPCs per animal that had adopted 1° or 2° vulval fates. Number of animals scored is designated by *n*. Alleles used: *pry-1(mu38), rom-1(zh18), rom-2(ok996), lin-12(n137gf), dpy-19(e1259), huIs7[hs::bar-1*Δ*NT], let-60(n1046gf), gaIs36[hs::mpk-1, D-mek-2(gf)], lin-15(n309), zhEx22[lin-3(+), sur-5::gfp, unc-119(+)],* and *syIs12[hs::lin-3extra].* Statistical analysis was done as described in Materials and Methods

^a^ Five independent transgenic lines generated with this construct displayed induction indices ranging from 4.1 to 5.4, and one of the lines displaying a penetrant Muv phenotype (*zhEx22*) was used for the further analysis in the different backgrounds

^b^ dsRNA was injected into the syncytial gonad of the parents, and vulval induction in the F1 progeny was scored for rows 4 and 11 by inspection under a dissecting microscope

^c^ To provide a low dose of LIN-3extra, L1 larvae were heat-shocked for 5 min at 33 °C and grown at 25 °C until L4

^d^ To provide a high dose of LIN-3extra, or MPK-1 or BAR-1ΔNT, respectively, early L21 larvae were heat-shocked for 30 min at 33 °C and grown at 25 °C until L4

^e^ These strains carried the *dpy-19(e1259)* mutation in cis to *lin-12(n137gf)*

** *p* ≤ 0.001; *** *p* ≤ 0.0001; numbers in brackets next to asterisks indicate the row to which a dataset was compared

n.d., no data

### ROM-1 Positively Regulates the EGFR/RAS/MAPK Pathway in Distal VPCs

Next, we examined whether loss of *rom-1* or *rom-2* function affects vulval induction in a sensitized genetic background by using mutations that hyperactivate the EGFR/RAS/MAPK pathway. The *rom-1(0)* mutation as well as *rom-1* RNAi partially suppressed the multivulva (Muv) phenotype caused by overexpression of the LIN-3 EGF growth factor *[lin-3(+)]* (Hill and Sternberg 1992) or by the *n1046* gain-of-function *(gf)* mutation in the *let-60 ras* gene, which renders vulval development partially independent of upstream signaling (Beitel et al. 1990; Chang et al. 2000) (Table 1, rows 5–7 and 12–14). In addition, the *rom-1(0)* mutation suppressed the Muv phenotype of *hs::mpk-1* animals that overexpress the wild-type MAPK MPK-1 under control of a heat-shock promoter together with *Drosophila* MEK-2 under control of the interferon-1α promoter (Lackner and Kim 1998) (Table 1, rows 16 and 17). In contrast to *rom-1,* neither the *rom-2(rf)* mutation nor *rom-2* RNAi affected the Muv phenotype of *let-60(gf)* animals (Table 1, row 15; unpublished data).

The *rom-1(0)* mutation did not significantly enhance the vulvaless (Vul) phenotype caused by the *lin-3(e1417), lin-2(n397), sem-5(n2019),* or *let-60(n2021)* mutations that reduce the activity of the receptor tyrosine kinase (RTK)/RAS/MAPK pathway (unpublished data). Since these Vul mutants affect the cell fates of only the proximal VPCs (P5.p, P6.p, and P7.p), ROM-1 plays no role in the induction of the proximal VPCs by the AC. Thus, ROM-1 enhances the activity of the EGFR/RAS/MAPK pathway to allow the induction of the distal VPCs P3.p, P4.p, and P8.p.

### ROM-1 Regulates LIN-3 EGF Activity during Vulval Induction

A soluble form of LIN-3 that consists of the extracellular domain with the EGF repeat but lacks the transmembrane and intracellular domains is biologically active and causes a Muv phenotype when overexpressed under control of a heat-shock promoter *(hs::lin-3extra)* (Katz et al. 1995). Unlike full-length LIN-3, the Muv phenotype induced by a low or high dosage of LIN-3extra was not suppressed by *rom-1(0)* (Table 1, rows 8–11). In *lin-15(rf)* mutants, all VPCs adopt vulval cell fates independently of the LIN-3 signal, though induction in *lin-15(rf)* mutants depends on the activity of LET-23 and the other components of the EGFR/RAS/MAPK pathway (Clark et al. 1994; Huang et al. 1994). The *rom-1(0)* mutation did not suppress the Muv phenotype of *lin-15(rf)* animals, suggesting that loss of *rom-1* function affects the LIN-3-dependent induction of vulval cell fates rather than the LIN-3-independent activity of the EGFR/RAS/MAPK pathway (Table 1, rows 18 and 19).

Finally, we examined the genetic interaction between *rom-1* and the Notch and Wnt pathways, since both pathways control vulval cell fate specification in parallel with the RTK/RAS/MAPK pathway (Wang and Sternberg 2001). In *lin-12 notch(gf)* animals, no AC is formed, and all VPCs adopt the 2° cell fate (Sternberg and Horvitz 1989). The same phenotype was observed in *rom-1(0) lin-12(gf)* double mutants (Table 1, rows 20 and 21). In addition, the Muv phenotype caused by hyperactivation of the Wnt pathway through a reduction-of-function mutation in *pry-1 axin* or by overexpression of a N-terminally truncated BAR-1 β-catenin protein was not suppressed by the *rom-1(0)* mutation (Table 1, rows 22–25) (Gleason et al. 2002).

In summary, these experiments suggest that ROM-1 promotes the LIN-3-dependent activation of the EGFR/RAS/MAPK signaling pathway. ROM-1 likely acts at the level or upstream of LIN-3 since full-length but not a soluble form of LIN-3 was sensitive to loss of *rom-1* function.

### ROM-1 Is Required to Transmit the Inductive Signal to the Distal VPCs

**Figure 2:**
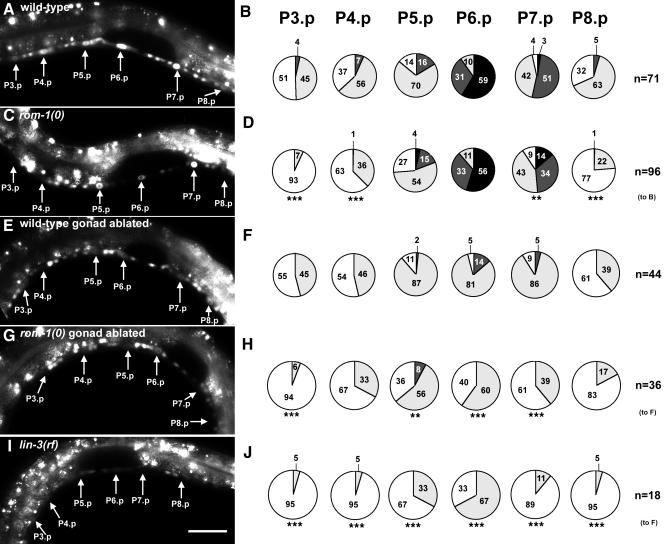
Expression of the *egl-17::cfp* Reporter in *rom-1(0)* and *lin-3(rf)* Mutants Photographic images on the left (A, C, E, G, and I) show the expression of the *arIs92[egl-17::cfp]* reporter in the VPCs of mid-L2 larvae of the different genotypes indicated. Pie graphs on the right (B, D, F, H, and J) show semi-quantitative representations of the expression levels observed in individual VPCs in the different backgrounds. A solid black color indicates the strongest expression of EGL-17::CFP as it was observed in P6.p of many (59%) wild-type animals; dark grey indicates intermediate, light grey weak, and white undetectable expression. The numbers inside the pie charts are the corresponding percentage values, and *n* refers to the number of animals examined for each case. EGL-17::CFP expression in each VPC of *rom-1(0)* or *lin-3(e1417rf)* animals was compared against the same VPC in wild-type animals (considered as expected value) with a Chi^2^ test for its independence; *** *p* ≤ 0.0001, ** *p* ≤ 0.001. The row to which a dataset was compared is indicated on the right. All photographs were taken with identical exposure and contrast settings. The scale bar in (I) is 20 μm.

To assess how much inductive signal each VPC receives, we examined the expression pattern of the *egl-17::cfp* reporter, which is a transcriptional target of the EGFR/RAS/MAPK pathway (Yoo et al. 2004). In mid L2 larvae, before the LIN-12 NOTCH-mediated lateral inhibition becomes effective, *egl-17::cfp* is expressed in a graded manner with highest levels in P6.p, intermediate levels in P5.p and P7.p (Yoo et al. 2004), and lower levels in P3.p, P4.p, and P8.p (Figure 2A and 2B). We therefore examined the effect of the *rom-1(0)* mutation on the *egl-17::cfp* expression pattern in mid L2 larvae. For this purpose, larvae were synchronized in the mid L1 stage at 13 h of development by letting them hatch in the absence of food, and then development was allowed to proceed by adding food for another 24 h until they reached the mid L2 stage (approximately 37 h of development). Loss of ROM-1 function had no effect on *egl-17::cfp* expression in the proximal VPCs (P5.p, P6.p, and P7.p), but significantly reduced *egl-17::cfp* expression in the distal VPCs when compared to wild-type animals (Figure 2C and 2D). Thus, ROM-1 increases the range of the inductive LIN-3 signal, allowing the distal VPCs to activate the EGFR/RAS/MAPK pathway. The altered *egl-17::cfp* expression pattern in *rom-1(0)* animals is consistent with the epistasis data, which showed that loss of *rom-1* function affects the induction of only the distal VPCs (see above).

### ROM-1 Is Expressed in the VPCs but Not in the AC during Vulval Induction

To analyze the expression pattern of ROM-1, we generated a transcriptional *rom-1* reporter by fusing 6.9 kb of the 5′ *rom-1* promoter/enhancer region to the *green fluorescent protein (gfp)* ORF carrying a nuclear localizing signal *(zhIs5[rom-1::nls::gfp])*. With a translational full-length *rom-1::gfp* fusion construct, we failed to obtain transgenic lines that consistently expressed ROM-1::GFP. Moreover, a genomic DNA fragment encompassing the entire *rom-1* locus failed to produce stable transgenic lines even when injected at relatively low concentrations (1–10 ng/μl), suggesting that elevated levels of ROM-1 are toxic to the animals.

**Figure 3:**
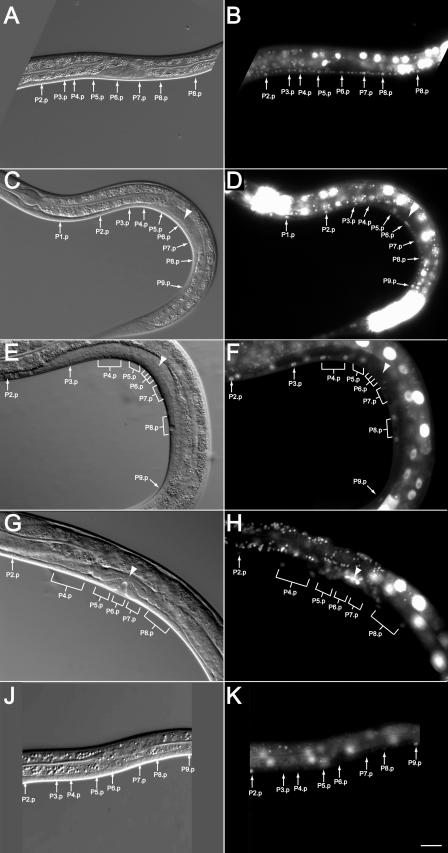
Expression Pattern of *rom-1::nls::gfp* Expression pattern of the *zhIs5[rom-1::nls::gfprom-1::]* transcriptional reporter during vulval development. Images on the left (A, C, E, G, and I) show the corresponding Nomarski pictures with the arrows pointing at the Pn.p cell nuclei and the arrowhead indicating the position of the AC nucleus. (B) A mid L2 larva before vulval induction with uniform *rom-1::nls::gfp* expression in all the Pn.p cells. (D) An early L3 larva in which *rom-1::nls::gfp* expression was decreased in all VPCs except P6.p (see text for a quantification of the expression pattern). Note that the nuclei of hyp7 and the Pn.p cells that had fused to hyp7 displayed strong *rom-1::nls::gfp* expression (P1.p, P2.p, P3.p and P9.p in the example shown). (F) A mid to late L3 larva in which P6.p had generated four descendants. Expression of *rom-1::nls::gfp* occurred only in the 3° descendants of P.4.p and P8.p after they fused to hyp7. (H) An L4 larva during vulval invagination. No *rom-1::nls::gfp* was detectable in the 1° and 2° descendants of P5.p, P6.p, and P7.p, but the AC and the surrounding uterine cells displayed strong *rom-1::nls::gfp* expression. (K) A late L2 to early L3 larva following the ablation of the precursors of the somatic gonad. No up-regulation of *rom-1::nls::gfp* in P5.p, P6.p, or P7.p was observed. The scale bar in (K) is 10 μm.

The transcriptional *rom-1::nls::gfp* reporter was widely expressed in somatic cells throughout development. Surprisingly, we did not detect any *rom-1::nls::gfp* expression in the gonadal AC before the L4 stage, while consistent expression was observed in the Pn.p cells and the Pn.a-derived neurons from the L1 stage on. In early L2 *zhIs5* larvae, the six VPCs expressed *rom-1::nls::gfp* at equal levels (Figure 3A and 3B). The Pn.p cells that are not part of the vulval equivalence group and had fused to hyp7 at the end of the L1 stage showed relatively higher *rom-1::nls::gfp* expression than the VPCs (for example, P1.p, P2.p, and P9.p in Figure 3C and 3D). Toward the end of the L2 stage, *rom-1::nls::gfp* expression decreased in distal VPCs adopting the 3° uninduced fate and persisted in the proximal VPCs adopting induced vulval fates (Figure 3C and 3D). In 60% of *zhIs5* animals, we observed an up-regulation of *rom-1::nls::gfp* in P6.p, and in 35% and 45% of the cases, *rom-1::nls::gfp* expression was higher in P5.p and P7.p, respectively (*n* = 20). After vulval induction, *rom-1::nls::gfp* was down-regulated in the 1° and 2° descendants of P5.p, P6.p and P7.p, while the 3° descendants of P3.p, P4.p, and P8.p again expressed high levels of *rom-1::nls::gfp* after they had fused with hyp7 (Figure 3E and 3F). Expression of *rom-1::nls::gfp* was observed in the AC and other cells of the somatic gonad only beginning in the L4 stage, before the AC fused with the uterine seam cell and persisting after fusion (Figure 3G and 3H) (Sulston and Horvitz 1977; Newman et al. 1996). To test whether the inductive AC signal is required for the elevated *rom-1::nls::gfp* expression in the proximal VPCs, we ablated in *zhIs5* animals the precursors of the somatic gonad Z1 and Z4 (Kimble 1981). Uniformly low *rom-1::nls::gfp* expression was found in all six VPCs of gonad-ablated *zhIs5* animals at the late L2 to early L3 stage, before the descendants of the 3° VPCs had fused to hyp7 (Figure 3J and 3K, *n* = 20). To test whether *rom-1::nls::gfp* expression depends on RTK/RAS/MAPK signaling in the VPCs, we introduced the *zhIs5* transgene into *lin-7(e1413)* mutants that exhibit a penetrant Vul phenotype due to reduced LET-23 EGFR activity (Simske et al. 1996). In *lin-7(e1413); zhIs5* animals, the up-regulation of *rom-1::nls::gfp* occurred less frequently (in 13%, 33%, and 7% of the cases in P5.p, P6.p, and P7.p, respectively, *n* = 15). Thus, the AC signal up-regulates *rom-1::nls::gfp* expression in the VPCs that adopt vulval cell fates.

### ROM-1 Acts in an AC-Independent Pathway that Promotes Vulval Induction

To examine whether ROM-1 acts in cells other than the AC (which is part of the somatic gonad), we tested the effect of loss of *rom-1(+)* function on vulval induction in gonad-ablated animals. If ROM-1 acts exclusively in the AC, then the *rom-1(0)* mutation should not affect vulval induction in gonad-ablated animals. On the other hand, if ROM-1 acts in cells other than the AC, then the *rom-1(0)* mutation should suppress vulval induction even in the absence of the AC. Since the inductive AC signal is absolutely required to initiate vulval development (Kimble 1981), we performed the gonad ablation experiments in *let-60(gf)* or *hs::mpk-1* animals that exhibit a hyperactive EGFR/RAS/MAPK signaling pathway causing AC-independent vulval induction (Table 2, rows 5 and 8) (Beitel et al. 1990; Lackner and Kim 1998; Chang et al. 2000). In addition, we examined *lin-3(+)* animals because, as reported previously by Hill and Sternberg (1992), in animals that overexpress wild-type *lin-3* under control of its own promoter, some vulval differentiation could still be observed in the absence of the AC, pointing at an additional source of LIN-3 from the transgene in cells outside of the gonad (Table 2, row 2). Loss of *rom-1* function in gonad-ablated *lin-3(+), let-60(gf),* or *hs::mpk-1* animals caused a strong further reduction in vulval induction (Table 2, compare rows 2 with 3, 5 with 6, and 8 with 9). In contrast, vulval induction in gonad-ablated *lin-15(rf)* animals that exhibit *lin-3* independent vulval differentiation was not affected by the *rom-1(0)* mutation (Table2, rows 13 and 14).

**Table 2:**
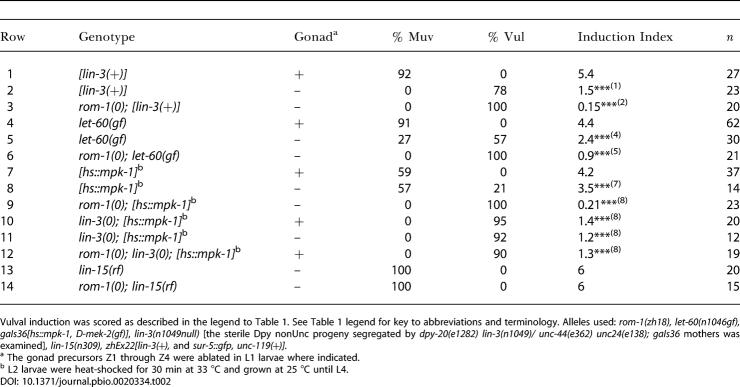
Gonad-Independent Function of *rom-1* and *lin-3*

Vulval induction was scored as described in the legend to Table 1. See Table 1 legend for key to abbreviations and terminology. Alleles used: *rom-1(zh18), let-60(n1046gf), gaIs36[hs::mpk-1, D-mek-2(gf)]*, *lin-3(n1049null)* [the sterile Dpy nonUnc progeny segregated by *dpy-20(e1282) lin-3(n1049)/ unc-44(e362) unc24(e138); gaIs36* mothers was examined], *lin-15(n309), zhEx22[lin-3(+),* and *sur-5::gfp, unc-119(+)].*

^a^ The gonad precursors Z1 through Z4 were ablated in L1 larvae where indicated

^b^ L2 larvae were heat-shocked for 30 min at 33 °C and grown at 25 °C until L4

Analogous results were obtained by examining the *egl-17::cfp* expression pattern after removal of the AC. In gonad-ablated animals, residual *egl-17::cfp* expression was observed in all VPCs (Figure 2E and 2F). In many cases, P6.p expressed higher levels of the reporter than did the other VPCs despite the absence of the AC. Loss of *rom-1* function in gonad-ablated animals caused a further decrease in *egl-17::cfp* expression in all VPCs (Figure 2G and 2H). Thus, ROM-1 acts in cells outside of the somatic gonad to promote vulval induction.

### An AC-Independent Activity of LIN-3 EGF

Next, we used an analogous strategy to test whether endogenous LIN-3 acts with ROM-1 in an AC-independent pathway. The decrease in vulval induction in *hs::mpk-1* animals that was caused by the *lin-3(n1049)* loss-of-function mutation *[lin-3(0)]* was much stronger than the decrease observed in gonad-ablated *lin-3(+); hs::mpk-1* animals (Table 2, compare rows 8 and 10; the L1 larval lethal phenotype caused by the *lin-3(0)* mutation was suppressed by the *hs::mpk-1* transgene). Vulval induction in *lin-3(0); hs::mpk-1* animals was not affected by gonad ablation since the *lin-3(0)* allele eliminated *lin-3* function in the AC (Table 2, compare rows 10 and 11). Thus, a complete loss of *lin-3* function had a more severe effect on vulval induction than did just the removal of the AC. Likewise, the *lin-3(e1417)* reduction-of-function mutation almost completely abolished the expression of the *egl-17::cfp* marker (Figure 2I and 2J). Thus, LIN-3 is also necessary for the AC-independent *egl-17::cfp* expression in the VPCs. Loss of *rom-1* function in a *lin-3(0); hs::mpk-1* background caused no further decrease in vulval induction, suggesting that ROM-1 does not affect vulval development in the absence of LIN-3 (Table 2, compare rows 10 and 12).

Taken together, these experiments indicate that not only the AC but also cells outside of the gonad produce LIN-3 to promote vulval fate specification. This AC-independent activity of LIN-3 requires ROM-1 function.

### ROM-1 Can Act in the Pn.p Cells

The absence of detectable *rom-1::nls::gfp* expression in the AC around the time of vulval induction and the AC-independent function of *rom-1* and *lin-3* suggested that *rom-1* may act cell-autonomously in the VPCs. To test this hypothesis, we expressed *rom-1* under control of the Pn.p cell-specific *lin-31* promoter *(lin-31::rom-1)* (Tan et al. 1998). The *lin-31::rom-1* transgene restored vulval induction in *rom-1(0); let-60(gf)* and *rom-1(0); hs::mpk-1* double mutants to levels comparable to those found in *let-60(gf)* and *hs::mpk-1* single mutants (Table 3, rows 1–3 and 8–10). A transgene encoding bacterial Cre recombinase under control of the *lin-31* promoter *(lin-31::cre)* that was used as a negative control had no effect on vulval induction (Table 3, rows 4 and 11) (Hoier et al. 2000). Consistent with a function of *rom-1* in an AC-independent pathway, the *lin-31::rom-1* transgene also increased induction in gonad-ablated *rom-1(0); let-60(gf)* animals (Table 3, rows 5–7). Finally, we expressed *rom-1* in the AC under control of the AC-specific enhancer (ACEL) *(ACEL::rom-1),* which is located in the third intron of the *lin-3* locus (Hwang and Sternberg 2003). In contrast to *lin-31::rom-1,* the *ACEL::rom-1* transgene did not rescue the suppression of the *let-60(gf)* Muv phenotype by *rom-1(0)* (Table 3, rows 12 and 13). Thus, the tissue-specific expression of ROM-1 in the Pn.p cells efficiently rescues a loss of *rom-1* function.

**Table 3:**
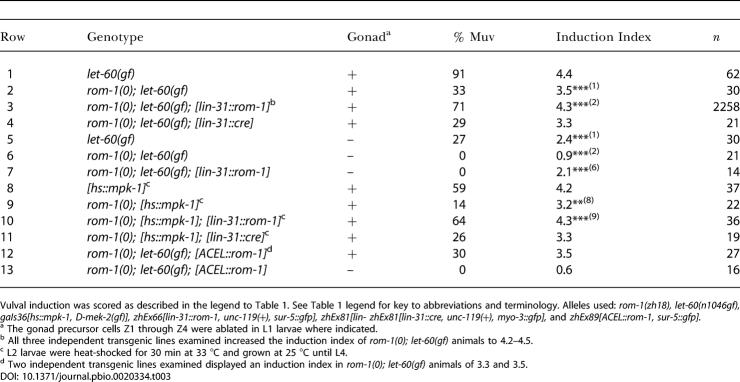
Expression of *rom-1* in the Pn.p Cells but Not in the AC Rescues the *rom-1(0)* Phenotype

Vulval induction was scored as described in the legend to Table 1. See Table 1 legend for key to abbreviations and terminology. Alleles used: *rom-1(zh18), let-60(n1046gf), gaIs36[hs::mpk-1, D-mek-2(gf)], zhEx66[lin-31::rom-1, unc-119(+), sur-5::gfp], zhEx81[lin- zhEx81[lin-31::cre, unc-119(+), myo-3::gfp],* and *zhEx89[ACEL::rom-1, sur-5::gfp].*

^a^ The gonad precursor cells Z1 through Z4 were ablated in L1 larvae where indicated

^b^ All three independent transgenic lines examined increased the induction index of *rom-1(0); let-60(gf)* animals to 4.2–4.5

^c^ L2 larvae were heat-shocked for 30 min at 33 °C and grown at 25 °C until L4

^d^ Two independent transgenic lines examined displayed an induction index in *rom-1(0); let-60(gf)* animals of 3.3 and 3.5

### LIN-3 EGF from the Pn.p Cells Amplifies the AC Signal

**Figure 4:**
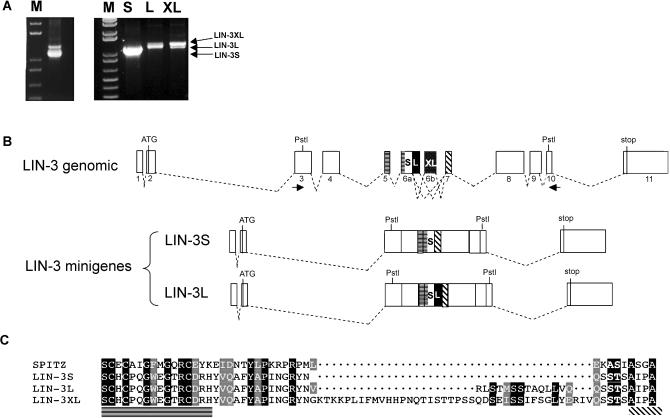
Alternative Splicing of *lin-3* mRNA (A) RT-PCR amplification of *lin-3* mRNA from mixed-stage N2 cDNA before (left) and after (right) size fractionation by preparative agarose gel electrophoresis. The lowest band corresponding to LIN-3S is most prominent, and the two upper bands correspond to LIN-3L and LIN-3XL. (B) Intron-exon structure of the *lin-3* locus. The *lin-3L* splice variant is generated by the usage of an alternative (more 3′ located) splice donor in exon 6a. The *lin-3XL* variant contains the additional exon 6b inserted between exons 6a and 7. The regions encoding the EGF repeat in exon 5 and part of 6a and the transmembrane domain in exon 7 are outlined, and the positions of the PstI sites used for the construction of the minigenes are indicated (see Materials and Methods). The structure of the *lin-3S* and *lin-3L* minigenes is shown in the lower part of the graphic. (C) Sequence alignment of the alternatively spliced region in LIN-3 with the corresponding region in *Drosophila* Spitz. The 15 and 41 amino acids in LIN-3L and LIN-3XL, respectively, in the juxtamembrane region break the alignment of LIN-3 with Spitz. The C-terminal end of the EGF domain is underlined with a horizontally hatched bar, and the beginning of the transmembrane domain is underlined by a diagonally hatched line.

To examine whether the VPCs or their descendants are the source of the AC-independent LIN-3 signal, we expressed *lin-3* dsRNA in the Pn.p cells in order to down-regulate by RNAi any possible *lin-3* expression in the VPCs (Timmons et al. 2003). For this purpose, a vector consisting of an inverted repeat of a 921-bp *lin-3* cDNA fragment under control of the same Pn.p cell-specific *lin-31* promoter used above *(lin-31::lin-3i)* was introduced into wild-type animals (Tan et al. 1998). Vulval induction occurred normally in *lin-31::lin-3i* animals (Table 4, row 1), although the adult animals displayed an 80% penetrant egg-laying defective (Egl) phenotype due to a defect in vulval morphogenesis (*n* = 122). In wild-type L4 larvae, the 1° descendants of P6.p in the vulF toroid ring (P6.papl/r and P6.ppal/r,), secrete LIN-3 to specify the ventral uterine (uv1) cell fate in the somatic gonad (Chang et al. 1999). If LIN-3 expression in the F cells is blocked through a mutation in the *egl-38 pax* transcription factor, then the uv1 cell adopts a uterine seam fate, resulting in an Egl phenotype. Thus, *lin-31::lin-3i* appeared to efficiently reduce LIN-3 expression in the vulval F cells without reducing the activity of LIN-3 in the AC. To further authenticate the efficiency of this approach, we crossed animals carrying the *lin-31::lin-3i* transgene to animals expressing the short splice variant of *lin-3* cDNA in the Pn.p cells under control of the *lin-31* promoter (*lin-31::lin-3S,* see below). The *lin-31::lin-3i* transgene almost completely suppressed the Muv phenotype caused by the *lin-31::lin-3S* transgene, while the *lin-31::cre* transgene that was used as negative control had no effect (Table 4, rows 2–4). Furthermore, the *lin-31::lin-3i* transgene significantly reduced vulval induction in *lin-3S* animals that carry a *lin-3* minigene encoding the short splice variant (Figure 4A), as well as in *let-60 ras(gf)* and *hs::mpk-1* animals (Table 4, rows 5–14)*.* Consistent with an AC-independent function of LIN-3 in the VPCs, the *lin-31::lin-3i* transgene also affected vulval induction in *let-60(gf)* animals lacking a gonad (Table 4, rows 11 and 12).

**Table 4:**
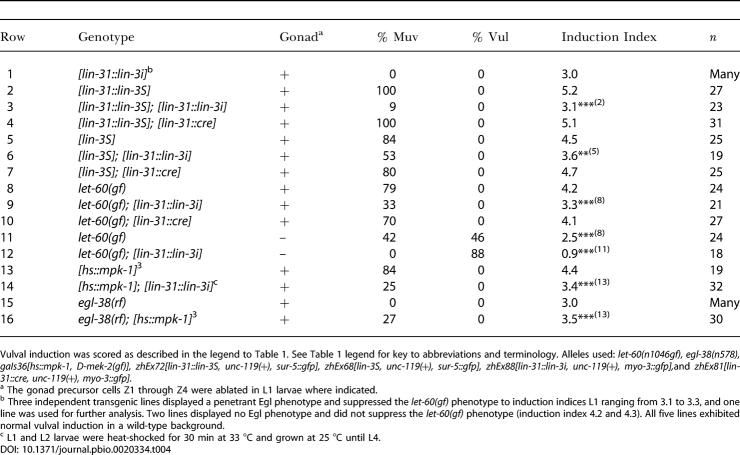
Pn.p Cell-Specific Function of *lin-3*

Vulval induction was scored as described in the legend to Table 1. See Table 1 legend for key to abbreviations and terminology. Alleles used: *let-60(n1046gf), egl-38(n578), gaIs36[hs::mpk-1, D-mek-2(gf)], zhEx72[lin-31::lin-3S, unc-119(+), sur-5::gfp], zhEx68[lin-3S, unc-119(+), sur-5::gfp], zhEx88[lin-31::lin-3i, unc-119(+), myo-3::gfp],*and *zhEx81[lin-31::cre, unc-119(+), myo-3::gfp].*

^a^ The gonad precursor cells Z1 through Z4 were ablated in L1 larvae where indicated

^b^ Three independent transgenic lines displayed a penetrant Egl phenotype and suppressed the *let-60(gf)* phenotype to induction indices L1 ranging from 3.1 to 3.3, and one line was used for further analysis. Two lines displayed no Egl phenotype and did not suppress the *let-60(gf)* phenotype (induction index 4.2 and 4.3). All five lines exhibited normal vulval induction in a wild-type background

^c^ L1 and L2 larvae were heat-shocked for 30 min at 33 °C and grown at 25 °C until L4

As an independent test to determine if a reduction of LIN-3 expression in the vulval cell lineage affects induction, we used the *n578* reduction-of-function mutation in the *egl-38 pax* transcription factor, because this *egl-38* allele has been shown to eliminate LIN-3 expression in the 1° cell lineage (Chang et al. 1999). Although *egl-38(rf)* single mutants exhibited wild-type levels of vulval induction, the *egl-38(rf)* mutation reduced the Muv phenotype of *hs::mpk-1* animals to a similar degree as the *rom-1(0)* mutation or the *lin-31::lin-3i* transgene (Table 4, rows 15 and 16). Thus, EGL-38 is necessary for the AC-independent function of LIN-3.

In summary, these experiments indicated that, during the process of vulval cell fate specification, some of the Pn.p cells (probably the VPCs) produce LIN-3 to amplify the inductive signal.

### Three LIN-3 EGF Splice Variants that Differ in the Juxtamembrane Domain

The *lin-3* locus encodes two splice variants termed LIN-3S (short) and LIN-3L (long) that are generated by the differential choice of the splice donor of exon 6 (Figure 4B) (Hill and Sternberg 1992). While performing RT-PCR experiments using a primer pair flanking the differentially spliced exons, we discovered a third splice variant, termed LIN-3XL, that is generated by the insertion of an additional exon (6b) between exons 6 and 7 (Figure 4A and 4B). The LIN-3XL splice variant was independently isolated from the yk1053b07EST clone. LIN-3XL contains a 41 amino acid insert, and LIN-3L contains a 15 amino acid insert, in the region between the EGF repeat and the transmembrane domain, when compared to LIN-3S (Figure 4C). Since the analogous region in *Drosophila* Spitz EGF is required for the proteolytic processing of Spitz by Rhomboid (Bang and Kintner 2000; Lee et al. 2001; Urban and Freeman 2003), we sought to determine which of the LIN-3 splice variants depend on ROM-1 activity. To address this question, we first constructed *lin-3* minigenes by replacing the differentially spliced exons with cDNA fragments encoding either of the splice forms (see Figure 4B). Both *lin-3L* and *lin-3S* minigenes were capable of inducing a Muv phenotype, but we observed a marked difference in the dosages required to elicit this phenotype. All (12 out of 12) transgenic lines generated by injection of a relatively low (1 ng/μl) or high (100 ng/μl) concentration of the *lin-3S* minigene exhibited a strong Muv phenotype (with induction indices ranging from 4.1 to 5.6). In contrast, the *lin-3L* construct caused a Muv phenotype only when injected at a high concentration. (None of the seven *lin-3L* lines obtained by injecting 1 ng/μl exhibited a Muv phenotype, while all nine lines obtained by injecting 100 ng/μl exhibited a Muv phenotype, with induction indices ranging from 4.2 to 5.0). For the *lin-3XL* construct, we obtained variable results; some lines exhibited a weak Muv and others no or even a Vul phenotype (unpublished data). Since we failed to observe a consistent phenotype with this minigene construct, we did not further pursue the analysis of the *lin-3XL* minigene.

### ROM-1 Is Necessary for the Activation of the Long LIN-3 Splice Variant

To investigate the genetic interactions between *rom-1* and the *lin-3* splice variants, we compared one line for each of the *lin-3S* and *lin-3L* minigenes that displayed a similar degree of vulval induction (Table 5, rows 1 and 4). Since the presence of endogenous LIN-3 might mask a specific requirement of either LIN-3 splice variant, we introduced the two minigenes into a *lin-3(0)* background. The *lin-3S* and *lin-3L* transgenes both rescued the larval lethality of *lin-3(0)* mutants, yielding adult Muv animals (Table 5, rows 2 and 5 and Table 6, rows 1 and 3). Since we used multicopy arrays that are silenced in the germ cells and LIN-3 is required in the oocytes to induce ovulation (Clandinin et al. 1998), the rescued *lin-3(0); lin-3S* and *lin-3(0); lin-3L* animals were sterile. Loss of *rom-1* function did not affect the viability or the Muv phenotype of *lin-3(0); lin-3S* animals (Table 5, rows 2 and 3 and Table 6, row 2). In contrast, the efficiency of the *lin-3L* transgene in rescuing the larval lethality of *lin-3(0)* mutants was reduced by loss of *rom-1* function (Table 6, row 4). Moreover, the rare *rom-1(0), lin-3(0); lin-3L* animals that escaped the larval lethality exhibited a weaker Muv phenotype than *lin-3(0); lin-3L* animals, suggesting that the function of LIN-3L during vulval induction partially depends on ROM-1 activity (Table 5, rows 5 and 6).

**Table 5:**
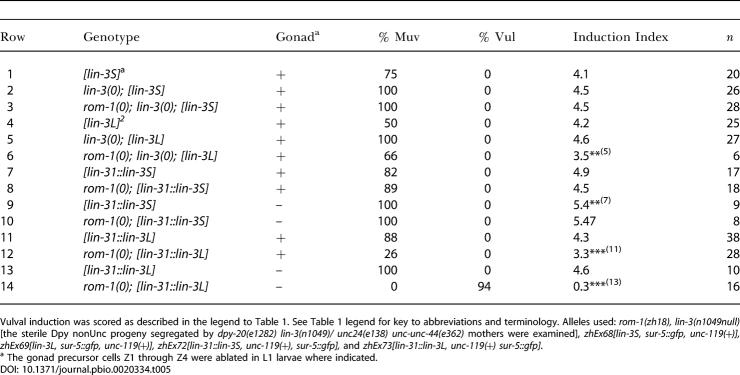
The *lin-3L* Splice Form Depends on *rom-1* Activity

Vulval induction was scored as described in the legend to Table 1. See Table 1 legend for key to abbreviations and terminology. Alleles used: *rom-1(zh18), lin-3(n1049null)* [the sterile Dpy nonUnc progeny segregated by *dpy-20(e1282) lin-3(n1049)/ unc24(e138) unc-unc-44(e362)* mothers were examined], *zhEx68[lin-3S, sur-5::gfp, unc-119(+)], zhEx69[lin-3L, sur-5::gfp, unc-119(+)], zhEx72[lin-31::lin-3S, unc-119(+), sur-5::gfp],* and *zhEx73[lin-31::lin-3L, unc-119(+) sur-5::gfp]*

^a^ The gonad precursor cells Z1 through Z4 were ablated in L1 larvae where indicated

**Table 6:**
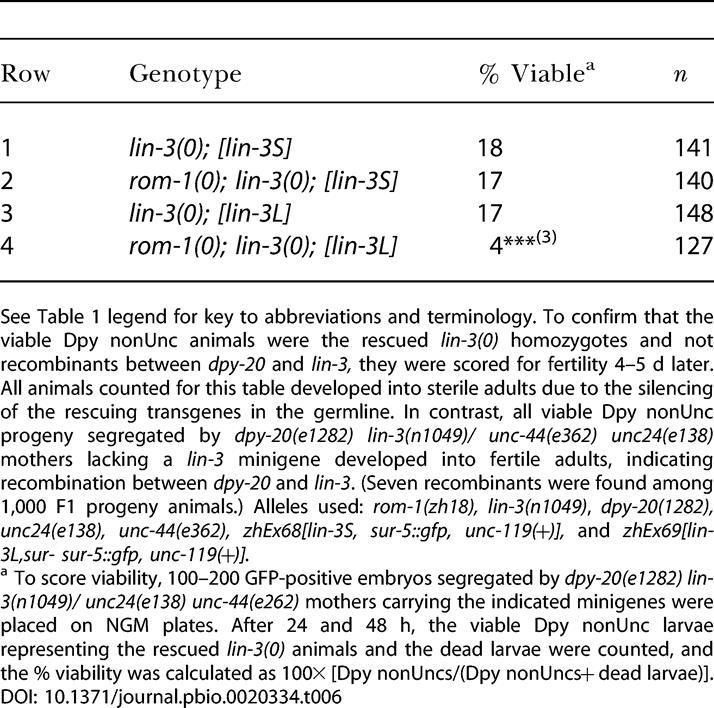
Rescue of the Larval Lethality in *lin-3(0)* Mutants by the *lin-3S* and *lin-3L* Minigenes

See Table 1 legend for key to abbreviations and terminology. To confirm that the viable Dpy nonUnc animals were the rescued *lin-3(0)* homozygotes and not recombinants between *dpy-20* and *lin-3,* they were scored for fertility 4–5 d later. All animals counted for this table developed into sterile adults due to the silencing of the rescuing transgenes in the germline. In contrast, all viable Dpy nonUnc progeny segregated by *dpy-20(e1282) lin-3(n1049)/ unc-44(e362) unc24(e138)* mothers lacking a *lin-3* minigene developed into fertile adults, indicating recombination between *dpy-20* and *lin-3*. (Seven recombinants were found among 1,000 F1 progeny animals.) Alleles used: *rom-1(zh18), lin-3(n1049)*, *dpy-20(1282), unc24(e138), unc-44(e362), zhEx68[lin-3S, sur-5::gfp, unc-119(+)],* and *zhEx69[lin-3L,sur- sur-5::gfp, unc-119(+)].*

^a^ To score viability, 100–200 GFP-positive embryos segregated by *dpy-20(e1282) lin-3(n1049)/ unc24(e138) unc-44(e262)* mothers carrying the indicated minigenes were placed on NGM plates. After 24 and 48 h, the viable Dpy nonUnc larvae representing the rescued *lin-3(0)* animals and the dead larvae were counted, and the % viability was calculated as 100× [Dpy nonUncs/(Dpy nonUncs+ dead larvae)]

To specifically test the function of the LIN-3 splice variants in the Pn.p cells, we cloned full-length cDNAs encoding the LIN-3S and LIN-3L splice variants under control of the Pn.p cell-specific *lin-31* promoter *(lin-31::lin-3S* and *lin-31::lin-3L)*. The *lin-31::lin-3S* and *lin-31::lin-3L* transgenes both caused a strong Muv phenotype in the presence and absence of the AC (Table 5, rows 7, 9, 11, and 13). Loss of *rom-1* function did not change the phenotype of *lin-31::lin-3S* animals (Table 5, rows 8–10), but it strongly suppressed the Muv phenotype of *lin-31::lin-3L* animals (Table 5, rows 12 and 14). Thus, ROM-1 is required for the activity of the LIN-3L splice variant in the Pn.p cells, while LIN-3S functions independently of ROM-1.

## Discussion

### ROM-1 Positively Regulates LIN-3 EGF Mediated Vulval Induction

Of the five Rhomboid proteins predicted by the complete C. elegans genome sequence, only ROM-1, the closest homolog of *Drosophila* Rhomboid-1, possesses the hallmarks of a serine protease with an intact catalytic center (Urban et al. 2001). Here, we show that ROM-1 acts as a positive regulator of the EGFR/RAS/MAPK signaling pathway during vulval induction, as loss of *rom-1* function partially suppresses ectopic vulval induction caused by hyperactivation of the EGFR/RAS/MAPK pathway. Our epistasis analysis points at a role of ROM-1 in activating LIN-3 EGF. The activity of a soluble form of LIN-3 lacking the transmembrane and intracellular domains is completely independent of ROM-1 activity, but the activity of full-length LIN-3 EGF is sensitive to loss of ROM-1 function. Moreover, a mutation in *lin-15,* which renders vulval induction independent of LIN-3 activity, is not suppressed by loss of *rom-1* function, but mutations in LET-23 or downstream components of the EGFR/RAS/MAPK pathway efficiently suppress the *lin-15* Muv phenotype (Clark et al. 1994; Huang et al. 1994). Although ROM-1 enhances the activity of the inductive LIN-3 EGF signal, ROM-1 is not required for vulval induction under normal growth conditions. Loss of *rom-1* function does not enhance the Vul phenotype caused by mutations that reduce RTK/RAS/MAPK signaling in the proximal VPCs, indicating that ROM-1 is not required for the induction of the proximal VPCs by the AC. These observations point at the existence of another, yet unidentified protease that mediates the release of LIN-3 from the AC. Like vertebrate TGF-α, and unlike *Drosophila* Spitz, which absolutely depends on Rhomboid function, membrane-bound LIN-3 might be cleaved at the surface of the AC by an ADAM family metalloprotease (Peschon et al. 1998). Another possibility our experiments have not ruled out is that in *rom-1(0)* mutants an unprocessed, membrane-bound form of LIN-3 that is retained on the plasma membrane of the AC induces the 1° fate in the adjacent VPC P6.p through juxtacrine signaling (Anklesaria et al. 1990). Once the AC has induced the 1° cell fate in P6.p, the 2° cell fate specification in the neighboring VPCs P5.p and P7.p can occur exclusively through lateral LIN-12 NOTCH signaling, resulting in a wild-type vulva (Greenwald et al. 1983; Kenyon 1995; Simske and Kim 1995). However, the AC is separated from the VPCs by two adjacent basal laminas that dissolve only after the vulval cell fates have been induced (Sherwood and Sternberg 2003). It is therefore difficult to predict whether LIN-3 anchored in the plasma membrane of the AC could reach its receptor LET-23 EGFR on the basolateral surface of P6.p.

### ROM-1 Is Required for LIN-3 EGF Activity in the Pn.p Cells

Three lines of evidence indicate that ROM-1 functions in the signal-receiving VPCs rather than the signal-sending AC. First, a *rom-1::nls::gfp* transcriptional reporter is expressed in the VPCs but not in the AC around the time of vulval induction. The *rom-1* gene appears to be a transcriptional target of the EGFR/RAS/MAPK pathway, as *rom-1::nls::gfp* expression is up-regulated in the induced VPCs in response to AC signaling. Second, the expression of *rom-1* in the Pn.p cells rescues loss of *rom-1* function. Third, loss of *rom-1* function in animals lacking an AC results in a further suppression of vulval induction and reduction of *egl-17::cfp* expression, indicating that the main, if not the only, focus of *rom-1* action is outside of the gonad. On the other hand, our epistasis analysis and the biochemical experiments done with *Drosophila* Rhomboid-1 (Bang and Kintner 2000; Lee et al. 2001; Urban and Freeman 2003) suggest that ROM-1 is required cell-autonomously for the activation of a membrane-bound LIN-3 precursor. This apparent discrepancy can be explained by the previously published observation of residual *lin-3* activity in the absence of the gonad (Hill and Sternberg 1992) and by the additional experiments presented in this paper that uncovered an AC-independent function of LIN-3 during vulval induction. The most likely source of LIN-3 besides the AC are the VPCs, as reducing *lin-3* function in the Pn.p cells by tissue-specific RNAi or a mutation in the *egl-38* gene, which is required for *lin-3* expression in vulval cells (Chang et al. 1999), had essentially the same effect as loss of *rom-1* function. Using transcriptional reporter constructs, *lin-3* expression has been observed in vulval cells of the 1° lineage beginning in the early L4 stage (Chang et al. 1999), and occasionally we observed weak *lin-3* expression in the VPCs or their daughter cells (unpublished data). It is possible that the reporter constructs used were lacking some of the regulatory sequences necessary to drive strong *lin-3* expression in the VPC lineage. Other potential sources of LIN-3 may be the posterior ectoderm or the excretory system in the head. However, it seems unlikely that LIN-3 secreted from cells at the anterior or posterior end of the animal influences vulval induction, since we did not observe a bias favoring the induction of anterior or posterior VPCs in the absence of the AC.

### A Relay Model for Vulval Induction

**Figure 5:**
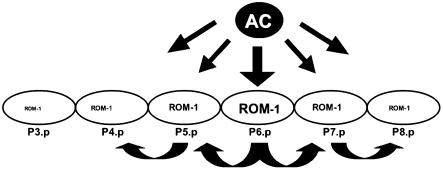
A Relay Model for Vulval Induction The AC initiates vulval development by secreting the LIN-3 growth factor independently of ROM-1. In response to the AC signal, the proximal VPCs up-regulate ROM-1 expression and start secreting LIN-3 in a ROM-1-dependent manner to relay the AC signal.

Expression levels of *rom-1::nls::gfp* are highest in the proximal VPCs (P5.p, P6.p, and P7.p) that adopt 1° and 2° vulval cell fates, suggesting that the proximal VPCs are competent to secrete LIN-3 in response to the inductive AC signal. LIN-3 from the proximal VPCs may facilitate the induction of the more distally located VPCs by paracrine signaling (Figure 5). Such a relay model is reminiscent of the EGF signaling during *Drosophila* oogenesis (Freeman et al. 1992; Wasserman and Freeman 1998). The Gurken growth factor produced by the *Drosophila* oocyte initially activates the EGFR/RAS/MAPK pathway in the adjacent epithelial follicle cells on the dorsal side of the oocyte independently of Rhomboid. In response to the Gurken signal, the dorsal follicle cells secrete Spitz in a Rhomboid-dependent manner and activate the EGFR in the neighboring follicle cells by paracrine signaling, allowing the signal to spread along the dorsal follicle cell layer. In contrast to *Drosophila* oogenesis, signal spreading is not necessary for the development of a wild-type C. elegans vulva. The AC needs to induce only the nearest VPC, P6.p, since a 1° cell can specify the 2° fate in the neighboring cells exclusively through lateral LIN-12 NOTCH signaling (Greenwald et al. 1983; Simske and Kim 1995). On the other hand, dosage experiments have indicated that low levels of inductive LIN-3 signal can directly specify the 2° cell fate in the absence of lateral signaling (Katz et al. 1995). It is therefore possible that the relay signal generated by ROM-1 and LIN-3 in P6.p contributes to the specification of the 2° cell fate in the neighboring VPCs in combination with the lateral LIN-12 NOTCH signal. LIN-3 secreted from the proximal VPCs could initially serve to maintain the competence of all VPCs, while at a later phase of induction the AC and lateral signals would seal the 1° fate of P6.p and the 2° fate of P5.p and P7.p, respectively. A similar two-step model of vulval induction has been proposed for other rhabditid nematode species such as *Oscheius* sp. (Felix et al. 2000). In *C. elegans,* ROM-1 is dispensable for the induction of the proximal VPCs, and the relay mechanism mediated by LIN-3 and ROM-1 only becomes apparent in a sensitized genetic background in which distal VPCs adopt induced cell fates. It is interesting to note in this context that in *Mesorhabditis* and *Teratorhabditis,* in which the vulva develops in the posterior body region, induction occurs without a signal from an AC or any other gonad cell (Sommer and Sternberg 1994). In these posterior-vulva Rhabditidae, the VPCs are not equivalent, because only P5.p and P6.p are competent to adopt the 1° fate. The mechanism that generates this intrinsic difference among the VPCs is unknown. It is possible that in these nematodes the specification of the VPC cell fates occurs in a cell-autonomous manner that could involve EGF signaling between the VPCs. The AC in C. elegans serves to position the vulva in the central body region, and the function of an AC appears to be absent in the posterior-vulva *Rhabditidae*.

### Splice Variant-Specific Action of ROM-1

The two previously identified LIN-3 splice forms (LIN-3S and LIN-3L) as well as the newly identified longer variant (LIN-3XL) differ by 15 and 41 amino acid insertions in the juxtamembrane region just prior to the predicted Rhomboid cleavage site at the start of the transmembrane domain (Hill and Sternberg 1992). Our experiments with LIN-3 minigenes indicate that the activity of the shortest splice form (LIN-3S) is completely independent of ROM-1 function. LIN-3S is expressed at all stages of development, and the LIN-3S minigene rescued all phenotypes caused by the *lin-3(0)* mutation, including the ovulation defects (unpublished data). This may explain why loss of *rom-1* function causes neither the larval lethality nor the sterility observed in *lin-3* mutants. Furthermore, our data indicate that LIN-3L function in the VPCs almost completely depends on ROM-1 activity. It seems improbable that the 15 amino acid insertion in LIN-3L could change the substrate specificity toward the ROM-1 protease. A more likely explanation for the inherent difference in the dependence of the LIN-3 splice variants on ROM-1 is suggested by the experiments performed with *Drosophila* Spitz (Lee et al. 2001; Tsruya et al. 2002). When expressed in mammalian cells that lack Rhomboid activity, Spitz is retained in the Golgi apparatus. Introducing functional Rhomboid into these cells allows the cleavage and release of Spitz from the Golgi apparatus, resulting in the secretion of the extracellular portion of Spitz. In analogy to Spitz, the small insert in LIN-3L could cause the retention of this LIN-3 isoform in the Golgi apparatus, thus rendering LIN-3L dependent on ROM-1 mediated processing. The tissue distribution of the LIN-3 splice variants is unknown, although all three forms can be detected by RT-PCR in L2 and L3 larvae around the time of vulval induction (unpublished data). In view of the relay model discussed above (Figure 5), tissue-specific splicing may account for the distinct functions of LIN-3. The roles of the two *Drosophila* EGF-like growth factors Gurken and Spitz may be fulfilled in C. elegans by the splice variants LIN-3S and LIN-3L, respectively. In this model, the AC uses the ROM-1-independent isoform LIN-3S to induce vulval development, and the proximal VPCs relay the AC signal to the distal VPCs by secreting LIN-3L. Alternative splicing has also been reported for the Neuregulin family of EGF-like ligands in vertebrates (Chang et al. 1997). Different Neuregulin isoforms elicit distinct responses by activating different EGFRs (Meyer et al. 1997). The tissue-specific expression of Rhomboid family proteases could determine which isoforms a particular cell type can secrete, thus adding another level of regulation.

## Materials and Methods

### 

#### General methods and strains used

Standard methods were used for maintaining and manipulating Caenorhabditis elegans (Brenner 1974). The C. elegans Bristol strain, variety N2, was used as the wild-type reference strain in all experiments. Unless noted otherwise, the mutations used have been described in Riddle and National Center for Biotechnology Information (U.S.) (2001) and are listed below by their linkage group: LGI: *pry-1(mu38)* (Gleason et al. 2002); LGIII: *dpy-19(e1259), lin-12(n137gf), rom-1(zh18)* (this study), *rom-2(ok966)* (C. elegans Gene Knockout Consortium), and *unc-119(e2498);* LGIV: *let-60(n1046)* (Beitel et al. 1990), *lin-3(n1049), unc-5(e53), unc-44(362), lin-45(sy96), unc-24(e138), mec-3(e1338), dpy-20(e1282), egl-38(n578),* and *mec-3(n3197);* LGX: *sem-5(n2019)* and *lin-15(n309);* extrachromosomal and integrated arrays: *zhEx22[lin-3(+), sur-5::gfp, unc-119(+)], zhE66[lin-31::rom-1, unc-119(+), sur-5::gfp], zhEx72[lin-31::lin-3S, unc-119(+), sur-5::gfp], zhEx68[lin-3S, unc-119(+), sur-5::gfp], zhEx69[lin-3L, sur-5::gfp, unc-119(+)],zhEx73[lin-31::lin- zhEx73[lin-31::lin-3L, unc-119(+) sur-5::gfp], zhEx78[ACEL::*Δ*pes-10::nls::gfp, unc-119(+)], zhEx81[lin-31::cre, unc-119(+), myo-3::gfp], zhE88[lin-31::lin-3i, unc-119(+), myo-3::gfp], zhEx89[ACEL::rom-1, sur-5::gfp], syIs12[hs::lin-3extra]* (Katz et al. 1995), *zhIs5[rom-1::nls::gfp, unc-119(+)]*, *huIs7[hs::bar-1*Δ*NT, dpy-20(+)]* (Gleason et al. 2002), *gaIs36[hs-mpk-1, IF1alpha-Dmek-2]* (Lackner and Kim 1998), and *arIs92[egl-17::cfp]* (Yoo et al. 2004). Unless noted in the table legends, all experiments were conducted at 20 °C. Transgenic lines were generated by injecting the experimental DNA at a concentration of 100 ng/μl or at the concentrations indicated in the text into both arms of the syncytial gonad as described (Mello et al. 1991).The constructs pUnc-119 (20 ng/μl), pPD93.97 (*myo-3::gfp*, 40 ng/μl), and pTG96 (*sur-5::gfp*, 100 ng/μl) were used as a cotransformation markers (Maduro and Pilgrim 1995; Yochem et al. 1997). The extrachromosomal array *zhEx[rom-1::nls::gfprom-1::gfp; unc-119(+)]* was integrated in the genome animals following γ-irradiation with 3,000 Rad to generate the array *zhIs5* and backcrossed six times before analysis. Double and triple mutants were constructed using standard genetic methods. Where cis-linked markers were used they are indicated in the table legends.

#### Plasmid constructs

The transcriptional *rom-1::nls::gfp* reporter construct (pRH2) was generated by ligating a HindIII-NheI–restricted 6,998-bp genomic fragment spanning the entire 5′ upstream region of F26F4.3 isolated by PCR amplification with the primers OAD49 (5′-GGAAGCTTGCATGCCCAACGAAATCGATA-3′) and OAD59 (5′-GGGCTAGCCATGTTGTGGAGAAGGAGAAC-3′) into the HindIII-XbaI site of pPD96.04. The *rom-1::gfp* translational reporter construct (pAD31) was generated by PCR amplification of a 3,146-bp genomic fragment containing 1,849 bp of 5′ sequences and the entire *rom-1* ORF using the primers OAD47 (5′-GACTCTAGAGTTGTCAAAAGGTCACGGG-3′) and OAD51 (5′-ATCCTCTAGAGTTGAGCAATTTTCGTTGTTCCAC-3′') followed by XbaI restriction and ligation to XbaI-digested vector pPD95.75. The upstream promoter region of this construct was further extended by replacing a 420-bp PstI fragment with a 2,099-bp PstI genomic fragment corresponding to positions –1,432 and –3,531 relative to the predicted translation start codon of F26F4.3. The *lin-31::rom-1* construct (pAD16) was generated by ligating a 1,601-bp SalI-NotI fragment spanning the entire *rom-1* coding sequence amplified with the primers OAD44 (5′-TTTTGGTCGACCTCCTTCTCCACAAC-3′) and OAD45 (5′-TTTGGCGGCCGCCTATGAGCAATTTTCG-3′) into the SalI-NotI site of the pB253 vector (Tan et al. 1998). To generate the *ACEL::rom-1* construct, a 2.3-kb SalI genomic *lin-3* fragment encompassing the third intron, which contains the ACEL (Hwang and Sternberg 2003), was cloned into the SalI site of the pTB11 plasmid, which consists of an *nls::gfp* reporter cassette under control of the truncated *pes-10* minimal promoter (Berset et al. 2001). Transgenic animals carrying the resulting *ACEL::*Δ*pes-10::nls::gfp* construct (pAH67) showed strong and specific GFP expression in the AC beginning in the mid L2 stage as reported (Hwang and Sternberg 2003). The KpnI-EcoRI fragment encoding the *nls::gfp* cassette was then replaced with a 1.6-kb KpnI-NotI *rom-1* fragment isolated from the *lin-31::rom-1* plasmid described above to yield the *ACEL::rom-1* construct. For the *lin-31::lin-3* splice variant constructs, partial cDNAs covering the differentially spliced region in the *lin-3* mRNA were amplified with the primers OAD31 (5′-CCCTTCGTGGTTTCGTCAAGAACGTAGTGC-3′) and OAD32 (5′-CGTATCTGCAGAATCCAACTCGATATTAATTAC-3′) using first-strand cDNA synthesized from mixed-stage total RNA as template. The PCR-amplified products were size-fractionated by agarose gel electrophoresis, cloned into the pGEMT vector (Promega), and sequenced to identify clones encoding individual splice variants. To obtain full-length *lin-3* cDNA construct (pAD27), a 1,996-bp XhoI fragment from the EST clone yk1053b07 (confirmed to encode full-length *lin-3XL* cDNA by DNA sequencing) was first subcloned into the XhoI site of a modified pBluescriptSK (Stratagene, La Jolla, California, United States) vector (pAD23) in which the PstI site had been destroyed by restriction with EcoRV and SmaI and religation of the resulting blunt ends. To generate full-length *lin-3S* and *lin-3L* cDNA constructs (pAD25 and pAD26, respectively), 1,065-bp and 1,110-bp PstI fragments specific for each splice variant isolated from the partial cDNA clones described above were used to replace the 1,188-bp PstI fragment in the full-length *lin-3XL* cDNA construct (pAD27). The *lin-31::lin-3S* and *lin-31::lin-3L* constructs were generated by cloning the 1,133-bp and 1,178-bp XhoI cDNA fragments of the S and L splice variants into the SalI site of pB253 (Tan et al. 1998). The *lin-3* splice variant minigene constructs (pAH63, pAH64, and pAH65 for *lin-3S, lin-3L,* and *lin-3XL,* respectively) were generated by cloning a 6.1-kb genomic fragment spanning the entire ORF of *lin-3* and 574 bp of 5′ and 236 bp of 3′ sequences amplified with the primers OAH137 (5′-CCAGAAAGTTCATGTGAATCAT-3′) and OAH138 (5′-TCACAGGAACTGAGAGGGAGAGTG-3′) into the pGEMT vector. From this construct, a 6,206-bp ApaI-SacI fragment was subcloned into pAD23 to obtain pAH62. The minigenes encoding each of the splice variants were obtained by replacing the 2,728-bp *lin-3* genomic PstI fragment with 1,065-, 1,110-, and 1,188-bp PstI fragments isolated from cDNAs of the different splice variants. To construct the *lin-31::lin-3* hairpin plasmid (pAD35), a 964-bp NdeI-HindIII *lin-3S cDNA* fragment from pAD25 was cloned into NdeI-HindIII–digested pAD27 using the recA^–^ E. coli SURE strain as host to obtain pAD32. The resulting 1,918-bp *lin-3* hairpin fragment was excised with XhoI from pAD32 and subcloned in E. coli SURE into the SalI site of the pB253 vector to obtain pAD35.

#### RNA interference

The dsRNA to interfere with *rom-1* function was generated by in vitro transcription using a 350-bp *rom-1* cDNA fragment corresponding to the nucleotides –17 to 725 relative to the predicted start codon of the ORF inserted into pGEM-T (Stratagene) as template. Transcripts were prepared using T7 and Sp6 RNA polymerase and annealed prior to injection as described (Fire et al. 1998). Progeny of the injected animals were assayed at 20 °C. RNAi of *rom-2* and *rom-3* was done by feeding the animals dsRNA-producing E. coli at 20 °C as described (Kamath et al. 2001).

#### Isolation of the *rom-1(zh18)* deletion allele

The *rom-1(zh18)* deletion mutant was isolated from an ethyl methanesulfonate–mutagenized library consisting of approximately 10^6^ haploid genomes as previously described (Jansen et al. 1997; Berset et al. 2001). DNA pools were screened by nested PCR with primers Rho13 (5′-GAGACCGGGGACCGTATTCTGGCAC-3′) and Rho10 (5′-GAGAGCATAAACTCCTGCGGAAGCACC-3′) in a first PCR reaction, and Rho35 (5′-GGGAATCCGACGGTGGTAGAAGC-3′) and Rho10 (5′-GAGAGCATAAACTCCTGCGGAAGCACC-3′) in a second PCR reaction. The *zh18* deletion removes 1,556 bp including 384 bp of 5′ upstream sequences and 618 bp of the *rom-1* ORF (positions 4906804–4908360 in the cosmid F26F4). The mutant strain was backcrossed six times against N2 before further experiments were done.

#### Vulval induction assay

Vulval induction was scored by examining worms at the L4 stage under Nomarski optics as described (Sternberg and Horvitz 1986). The number of VPCs that adopted a 1° or 2° vulval fate was counted for each animal as described (Sternberg and Horvitz 1986), and the induction index was calculated by dividing the number of 1° or 2° induced cells by the number of animals scored. Statistical analysis was performed using a t-test for independent samples. To remove the AC, the nuclei of the Z1 to Z4 gonadal precursor cells were ablated in early L1 larvae with a laser microbeam as described (Sulston and White 1980; Kimble 1981). The operated animals were allowed to develop until the L4 stage. Only those animals in which neither gonad arm developed and no residual gonadal cells survived were scored.

## Supporting Information

### Accession Numbers

The GenBank (http://www.ncbi.nlm.nih.gov/) accession numbers of the Rhomboid genes discussed in this paper are C.e. ROM-1 (AAA91218), C.e. ROM-2 (CAA82377), C.e. ROM-3 (CAB55154), C.e. ROM-4 (CAB55122), C.e. ROM-5 (AAF60768), D.m. Rho-1 (CAA36692), D.m. Rho-2 (AAK06752), D.m. Rho-3 (AAK06753), D.m. Rho-4 (AAK06754), D.m. Rho-6 (NP_523557), H.s. Rho-1 (CAA76629).

## Abbreviations

AC: anchor cell
ACEL: AC-specific enhancer
ADAM: a disintegrin and metalloprotease
EGF: epidermal growth factor
EGFR: EGF receptor
Egl: egg-laying defective
GFP: green fluorescent protein
hyp7: hypodermal syncytium
L[N]: larval stage [N]
MAPK: mitogen-activated protein kinase
Muv: multivulva
NLS: nuclear localization signal
ORF: open reading frame
1°: primary
RNAi: RNA interference
RTK: receptor tyrosine kinase
2°: secondary
3°: tertiary
TGF: transforming growth factor
VPC: vulval precursor cell
Vul: vulvaless

## References

[pbio-0020334-Anklesaria1] Anklesaria P, Teixido J, Laiho M, Pierce JH, Greenberger JS (1990). Cell-cell adhesion mediated by binding of membrane-anchored transforming growth factor alpha to epidermal growth factor receptors promotes cell proliferation. Proc Natl Acad Sci U S A.

[pbio-0020334-Arribas1] Arribas J, Coodly L, Vollmer P, Kishimoto TK, Rose-John S (1996). Diverse cell surface protein ectodomains are shed by a system sensitive to metalloprotease inhibitors. J Biol Chem.

[pbio-0020334-Bang1] Bang AG, Kintner C (2000). Rhomboid and Star facilitate presentation and processing of the *Drosophila* TGF-alpha homolog Spitz. Genes Dev.

[pbio-0020334-Beitel1] Beitel GJ, Clark SG, Horvitz HR (1990). Caenorhabditis elegans ras gene *let-60* acts as a switch in the pathway of vulval induction. Nature.

[pbio-0020334-Berset1] Berset T, Hoier EF, Battu G, Canevascini S, Hajnal A (2001). Notch inhibition of RAS signaling through MAP kinase phosphatase LIP-1 during C. elegans vulval development. Science.

[pbio-0020334-Bier1] Bier E, Jan LY, Jan YN (1990). *Rhomboid*, a gene required for dorsoventral axis establishment and peripheral nervous system development in Drosophila melanogaster. Genes Dev.

[pbio-0020334-Bogdan1] Bogdan S, Klambt C (2001). Epidermal growth factor receptor signaling. Curr Biol.

[pbio-0020334-Bosenberg1] Bosenberg MW, Pandiella A, Massague J (1993). Activated release of membrane-anchored TGF-alpha in the absence of cytosol. J Cell Biol.

[pbio-0020334-Brenner1] Brenner S (1974). The genetics of Caenorhabditis elegans. Genetics.

[pbio-0020334-Chang1] Chang C, Newman AP, Sternberg PW (1999). Reciprocal EGF signaling back to the uterus from the induced C. elegans vulva coordinates morphogenesis of epithelia. Curr Biol.

[pbio-0020334-Chang2] Chang C, Hopper NA, Sternberg PW (2000). Caenorhabditis elegans SOS-1 is necessary for multiple RAS-mediated developmental signals. EMBO J.

[pbio-0020334-Chang3] Chang H, Riese DJ 2nd, Gilbert W, Stern DF, McMahan UJ (1997). Ligands for ErbB-family receptors encoded by a neuregulin-like gene. Nature.

[pbio-0020334-Clandinin1] Clandinin TR, DeModena JA, Sternberg PW (1998). Inositol trisphosphate mediates a RAS-independent response to LET-23 receptor tyrosine kinase activation in C. elegans. Cell.

[pbio-0020334-Clark1] Clark SG, Lu X, Horvitz HR (1994). The Caenorhabditis elegans locus lin-15, a negative regulator of a tyrosine kinase signaling pathway, encodes two different proteins. Genetics.

[pbio-0020334-Felix1] Felix MA, De Ley P, Sommer RJ, Frisse L, Nadler SA (2000). Evolution of vulva development in the Cephalobina (Nematoda). Dev Biol.

[pbio-0020334-Fire1] Fire A, Xu S, Montgomery MK, Kostas SA, Driver SE (1998). Potent and specific genetic interference by double-stranded RNA in Caenorhabditis elegans. Nature.

[pbio-0020334-Fraser1] Fraser AG, Kamath RS, Zipperlen P, Martinez-Campos M, Sohrmann M (2000). Functional genomic analysis of C. elegans chromosome I by systematic RNA interference. Nature.

[pbio-0020334-Freeman1] Freeman M, Gurdon JB (2002). Regulatory principles of developmental signaling. Annu Rev Cell Dev Biol.

[pbio-0020334-Freeman2] Freeman M, Klambt C, Goodman CS, Rubin GM (1992). The *argos* gene encodes a diffusible factor that regulates cell fate decisions in the *Drosophila* eye. Cell.

[pbio-0020334-Gallio1] Gallio M, Sturgill G, Rather P, Kylsten P (2002). A conserved mechanism for extracellular signaling in eukaryotes and prokaryotes. Proc Natl Acad Sci U S A.

[pbio-0020334-Gleason1] Gleason JE, Korswagen HC, Eisenmann DM (2002). Activation of Wnt signaling bypasses the requirement for RTK/Ras signaling during C. elegans vulval induction. Genes Dev.

[pbio-0020334-Golembo1] Golembo M, Raz E, Shilo BZ (1996). The *Drosophila* embryonic midline is the site of Spitz processing, and induces activation of the EGF receptor in the ventral ectoderm. Development.

[pbio-0020334-Greenwald1] Greenwald IS, Sternberg PW, Horvitz HR (1983). The *lin-12* locus specifies cell fates in Caenorhabditis elegans. Cell.

[pbio-0020334-Guichard1] Guichard A, Roark M, Ronshaugen M, Bier E (2000). *Brother of rhomboid*, a rhomboid-related gene expressed during early *Drosophila* oogenesis, promotes EGF-R/MAPK signaling. Dev Biol.

[pbio-0020334-Hill1] Hill RJ, Sternberg PW (1992). The gene *lin-3* encodes an inductive signal for vulval development in C. elegans. Nature.

[pbio-0020334-Hoier1] Hoier EF, Mohler WA, Kim SK, Hajnal A (2000). The Caenorhabditis elegans APC-related gene *apr-1* is required for epithelial cell migration and *Hox* gene expression. Genes Dev.

[pbio-0020334-Huang1] Huang LS, Tzou P, Sternberg PW (1994). The *lin-15* locus encodes two negative regulators of Caenorhabditis elegans vulval development. Mol Biol Cell.

[pbio-0020334-Hwang1] Hwang BJ, Sternberg PW (2003). A cell-specific enhancer that specifies lin-3 expression in the C. elegans anchor cell for vulval development. Development.

[pbio-0020334-Jansen1] Jansen G, Hazendonk E, Thijssen KL, Plasterk RH (1997). Reverse genetics by chemical mutagenesis in Caenorhabditis elegans. Nat Genet.

[pbio-0020334-Kamath1] Kamath RS, Martinez-Campos M, Zipperlen P, Fraser AG, Ahringer J (2001). Effectiveness of specific RNA-mediated interference through ingested double-stranded RNA in Caenorhabditis elegans. Genome Biol.

[pbio-0020334-Katz1] Katz WS, Hill RJ, Clandinin TR, Sternberg PW (1995). Different levels of the C. elegans growth factor LIN-3 promote distinct vulval precursor fates. Cell.

[pbio-0020334-Kenyon1] Kenyon C (1995). A perfect vulva every time: Gradients and signaling cascades in C. elegans. Cell.

[pbio-0020334-Kimble1] Kimble J (1981). Alterations in cell lineage following laser ablation of cells in the somatic gonad of Caenorhabditis elegans. Dev Biol.

[pbio-0020334-Kornfeld1] Kornfeld K (1997). Vulval development in Caenorhabditis elegans. Trends Genet.

[pbio-0020334-Lackner1] Lackner MR, Kim SK (1998). Genetic analysis of the Caenorhabditis elegans MAP kinase gene *mpk-1*. Genetics.

[pbio-0020334-Lee1] Lee JR, Urban S, Garvey CF, Freeman M (2001). Regulated intracellular ligand transport and proteolysis control EGF signal activation in *Drosophila*. Cell.

[pbio-0020334-Maduro1] Maduro M, Pilgrim D (1995). Identification and cloning of *unc-119*, a gene expressed in the Caenorhabditis elegans nervous system. Genetics.

[pbio-0020334-McQuibban1] McQuibban GA, Saurya S, Freeman M (2003). Mitochondrial membrane remodelling regulated by a conserved rhomboid protease. Nature.

[pbio-0020334-Mello1] Mello CC, Kramer JM, Stinchcomb D, Ambros V (1991). Efficient gene transfer in *C.elegans*: Extrachromosomal maintenance and integration of transforming sequences. EMBO J.

[pbio-0020334-Meyer1] Meyer D, Yamaai T, Garratt A, Riethmacher-Sonnenberg E, Kane D (1997). Isoform-specific expression and function of neuregulin. Development.

[pbio-0020334-Newman1] Newman AP, White JG, Sternberg PW (1996). Morphogenesis of the C. elegans hermaphrodite uterus. Development.

[pbio-0020334-Pandiella1] Pandiella A, Massague J (1991). Cleavage of the membrane precursor for transforming growth factor alpha is a regulated process. Proc Natl Acad Sci U S A.

[pbio-0020334-Peschon1] Peschon JJ, Slack JL, Reddy P, Stocking KL, Sunnarborg SW (1998). An essential role for ectodomain shedding in mammalian development. Science.

[pbio-0020334-Riddle1] Riddle DL, National Center for Biotechnology Information (2001). C. elegans II.

[pbio-0020334-Rutledge1] Rutledge BJ, Zhang K, Bier E, Jan YN, Perrimon N (1992). The Drosophila spitz gene encodes a putative EGF-like growth factor involved in dorsal-ventral axis formation and neurogenesis. Genes Dev.

[pbio-0020334-Sherwood1] Sherwood DR, Sternberg PW (2003). Anchor cell invasion into the vulval epithelium in C. elegans. Dev Cell.

[pbio-0020334-Simske1] Simske JS, Kim SK (1995). Sequential signalling during Caenorhabditis elegans vulval induction. Nature.

[pbio-0020334-Simske2] Simske JS, Kaech SM, Harp SA, Kim SK (1996). LET-23 receptor localization by the cell junction protein LIN-7 during C. elegans vulval induction. Cell.

[pbio-0020334-Sommer1] Sommer RJ, Sternberg PW (1994). Changes of induction and competence during the evolution of vulva development in nematodes. Science.

[pbio-0020334-Sternberg1] Sternberg PW (1988). Lateral inhibition during vulval induction in Caenorhabditis elegans. Nature.

[pbio-0020334-Sternberg2] Sternberg PW, Han M (1998). Genetics of RAS signaling in C. elegans. Trends Genet.

[pbio-0020334-Sternberg3] Sternberg PW, Horvitz HR (1986). Pattern formation during vulval development in C. elegans. Cell.

[pbio-0020334-Sternberg4] Sternberg PW, Horvitz HR (1989). The combined action of two intercellular signaling pathways specifies three cell fates during vulval induction in C. elegans. Cell.

[pbio-0020334-Sulston1] Sulston JE, Horvitz HR (1977). Post-embryonic cell lineages of the nematode, Caenorhabditis elegans. Dev Biol.

[pbio-0020334-Sulston2] Sulston JE, White JG (1980). Regulation and cell autonomy during postembryonic development of Caenorhabditis elegans. Dev Biol.

[pbio-0020334-Tan1] Tan PB, Lackner MR, Kim SK (1998). MAP kinase signaling specificity mediated by the LIN-1 Ets/LIN-31 WH transcription factor complex during C. elegans vulval induction. Cell.

[pbio-0020334-Thomas1] Thomas JH, Stern MJ, Horvitz HR (1990). Cell interactions coordinate the development of the C. elegans egg-laying system. Cell.

[pbio-0020334-Thompson1] Thompson JD, Gibson TJ, Plewniak F, Jeanmougin F, Higgins DG (1997). The CLUSTAL_X windows interface: Flexible strategies for multiple sequence alignment aided by quality analysis tools. Nucleic Acids Res.

[pbio-0020334-Timmons1] Timmons L, Tabara H, Mello CC, Fire AZ (2003). Inducible systemic RNA silencing in Caenorhabditis elegans. Mol Biol Cell.

[pbio-0020334-Tsruya1] Tsruya R, Schlesinger A, Reich A, Gabay L, Sapir A (2002). Intracellular trafficking by Star regulates cleavage of the *Drosophila* EGF receptor ligand Spitz. Genes Dev.

[pbio-0020334-Urban1] Urban S, Freeman M (2002). Intramembrane proteolysis controls diverse signalling pathways throughout evolution. Curr Opin Genet Dev.

[pbio-0020334-Urban2] Urban S, Freeman M (2003). Substrate specificity of rhomboid intramembrane proteases is governed by helix-breaking residues in the substrate transmembrane domain. Mol Cell.

[pbio-0020334-Urban3] Urban S, Lee JR, Freeman M (2001). *Drosophila* rhomboid-1 defines a family of putative intramembrane serine proteases. Cell.

[pbio-0020334-Urban4] Urban S, Lee JR, Freeman M (2002). A family of Rhomboid intramembrane proteases activates all *Drosophila* membrane-tethered EGF ligands. Embo J.

[pbio-0020334-Wang1] Wang M, Sternberg PW (2001). Pattern formation during C. elegans vulval induction. Curr Top Dev Biol.

[pbio-0020334-Wasserman1] Wasserman JD, Freeman M (1998). An autoregulatory cascade of EGF receptor signaling patterns the *Drosophila* egg. Cell.

[pbio-0020334-Wasserman2] Wasserman JD, Urban S, Freeman M (2000). A family of rhomboid-like genes: *Drosophila* rhomboid-1 and roughoid/rhomboid-3 cooperate to activate EGF receptor signaling. Genes Dev.

[pbio-0020334-Whitfield1] Whitfield CW, Benard C, Barnes T, Hekimi S, Kim SK (1999). Basolateral localization of the Caenorhabditis elegans epidermal growth factor receptor in epithelial cells by the PDZ protein LIN-10. Mol Biol Cell.

[pbio-0020334-Wolfe1] Wolfe MS, De Los Angeles J, Miller DD, Xia W, Selkoe DJ (1999). Are presenilins intramembrane-cleaving proteases? Implications for the molecular mechanism of Alzheimer's disease. Biochemistry.

[pbio-0020334-Yochem1] Yochem J, Sundaram M, Han M (1997). Ras is required for a limited number of cell fates and not for general proliferation in Caenorhabditis elegans. Mol Cell Biol.

[pbio-0020334-Yoo1] Yoo AS, Bais C, Greenwald I (2004). Crosstalk between the EGFR and LIN-12/Notch pathways in C. elegans vulval development. Science.

